# Association of antimicrobial resistance and gut microbiota composition in human and non-human primates at an urban ecotourism site

**DOI:** 10.1186/s13099-020-00352-x

**Published:** 2020-03-10

**Authors:** C. W. Chong, A. H. S. Alkatheeri, N. Ali, Z. H. Tay, Y. L. Lee, S. J. Paramasivam, K. Jeevaratnam, W. Y. Low, S. H. E. Lim

**Affiliations:** 1 School of Pharmacy, Monash University Malaysia, Jalan Lagoon Selatan, 47500 Bandar Sunway, Selangor Darul Ehsan Malaysia; 2 Health Science Division, Abu Dhabi Women’s College, Higher Colleges of Technology, 41012 Abu Dhabi, UAE; 3 Royal College of Surgeons in Ireland, Perdana University, MAEPS Building, 43400 Serdang, Selangor Malaysia; 4 Faculty of Health and Medical Sciences, University of Surrey, Guildford, GU2 7AL UK; 5 Centre for Bioinformatics, School of Data Sciences, Perdana University, MAEPS Building, 43400 Serdang, Selangor Malaysia; 6 The Davies Research Centre, School of Animal and Veterinary Sciences, University of Adelaide, Roseworthy, SA 5371 Australia; 7 Animal Neighbours Project, School of Veterinary Medicine, Faculty of Health and Medical Sciences, University of Surrey, Guildford, GU2 7AL UK

**Keywords:** Ecotourism, Non-human primates, Human animal interaction, Antibiotic resistance

## Abstract

**Background:**

The rise of nature-based ecotourism in the past decade has introduced unprecedented challenges in managing the increasing interaction between humans and animals. The potential transmission of antibiotic resistant microbes between humans and non-human primate populations is a concern due to their genetic similarity. Malaysia is well known for hotspots of wildlife diversity where non-human primates like monkeys and orangutans have become popular tourist attractions. In this study, we assessed the prevalence of antimicrobial resistant *Staphylococcus aureus, Enterococcus* species, and other Enterobacteriaceae in the faeces of human (HS) and two non-human primates (NHP) in Malaysia, the Long-tailed macaque (*Macaca fascicularis,* MF) and Silvered leaf monkey (*Trachypithecus cristatus,* TC). In addition, the faecal bacterial composition was profiled to evaluate the potential association between antibiotic resistant profiles and composition of gut microbiota.

**Results:**

We tested the isolated bacteria using a selection of antibiotics. The results showed that both the number of antibiotic resistant strains and resistance level were higher in humans than NHPs. Overall, the composition of gut microbiome and pattern of antibiotic resistance showed that there was higher similarity between MF and TC, the two NHPs, than with HS. In addition, samples with higher levels of antibiotic resistance showed lower bacterial richness. *Homo sapiens* had the lowest bacterial diversity and yet it had higher abundance of *Bacteroides*. In contrast, NHPs displayed higher bacterial richness and greater prevalence of Firmicutes such as *Ruminococceae* and *Oscillospira.*

**Conclusion:**

Higher antibiotic susceptibility in NHPs is likely related to low direct exposure to antibiotics. The lack of resistance may also suggest limited antimicrobial resistance transmission between humans and NHP. Nonetheless, continued monitoring over a long period will help mitigate the risk of anthropozoonosis and zooanthroponosis.

## Background

Ecotourism is one of the fastest growing sectors in Malaysia. Since the formulation of the National Ecotourism Plan, the number of tourists visiting Malaysia had soared from 5.5 million in 1998 to 27.5 million in 2015 [1]. As the bulk of tourists comprises international travelers, close human-animal interaction such as feeding may facilitate zooanthroponosis transfer, leading to mortality in the wild animal population [2]. This is exacerbated by the fact that tourists generally lack the fundamental knowledge on the risk of pathogen transmission to animals [3].

Ecotourism is a growing sector in many countries due to increasing appreciation and desire to observe and interact with animals in their natural setting. It provides for a more up-close and personal contact between tourists and wild animals whilst experiencing nature simultaneously which are considered more enjoyable [4]. Subsidiary benefits of ecotourism include support of conservation for natural ecosystems and the promotion of sustainable local development [5]. Nevertheless, the lack of awareness about pathogen transmission amongst tourists as well as non-controlled interactions among visitors and wildlife (due to the poor management by ecotourism operators) pose a potential healthcare risks such as transmission of infectious diseases [2]. Additionally, the close interaction may increase the transmission of antibiotic resistant strains between tourists and wild animals [6]. Separately, the transfer of non-pathogenic bacteria between human and animal may alter the composition of microbiome in their gut [7], and as a consequence, affects the ability of the host to resist colonization of exogenous bacteria [8]. Conversely, sick animals may increase the risk of zooanthroponosis, and transferring infections to tourists [9].

Further to the above, human animal interaction (HAI) plays a role in altering the behavioural routines of wild animals. For example, consistent feeding from tourists can results in wild animals spending less time foraging for food, socializing with each other and a reduction in travel distance [10, 11]. Prolonged negative HAI is associated with more aggressive displays of behavior of animals towards tourists, culminating into cases of attack [12].

In this study, we focused on the non- human primates (NHP) from Kuala Selangor Nature Park as a non-conventional setting for transmission of antimicrobial resistance (AMR). We investigated NHPs in urban areas because they are known reservoir of zoonotic diseases to humans and are particularly popular at ecotourism destinations, especially within ASEAN. The aims of this study were to evaluate the prevalence of selected antibiotic-resistant bacteria in humans and in NHPs, in addition to understanding the relationship between gut bacterial composition and antibiotic resistant bacterial carriage rate of these hosts at an ecotourism site. To the best of our knowledge, this is the first report on antibiotic resistance and gut microbial composition of urban monkeys in Malaysia.

## Results

### Disk diffusion testing

#### MRSA

**Fig. 1:**
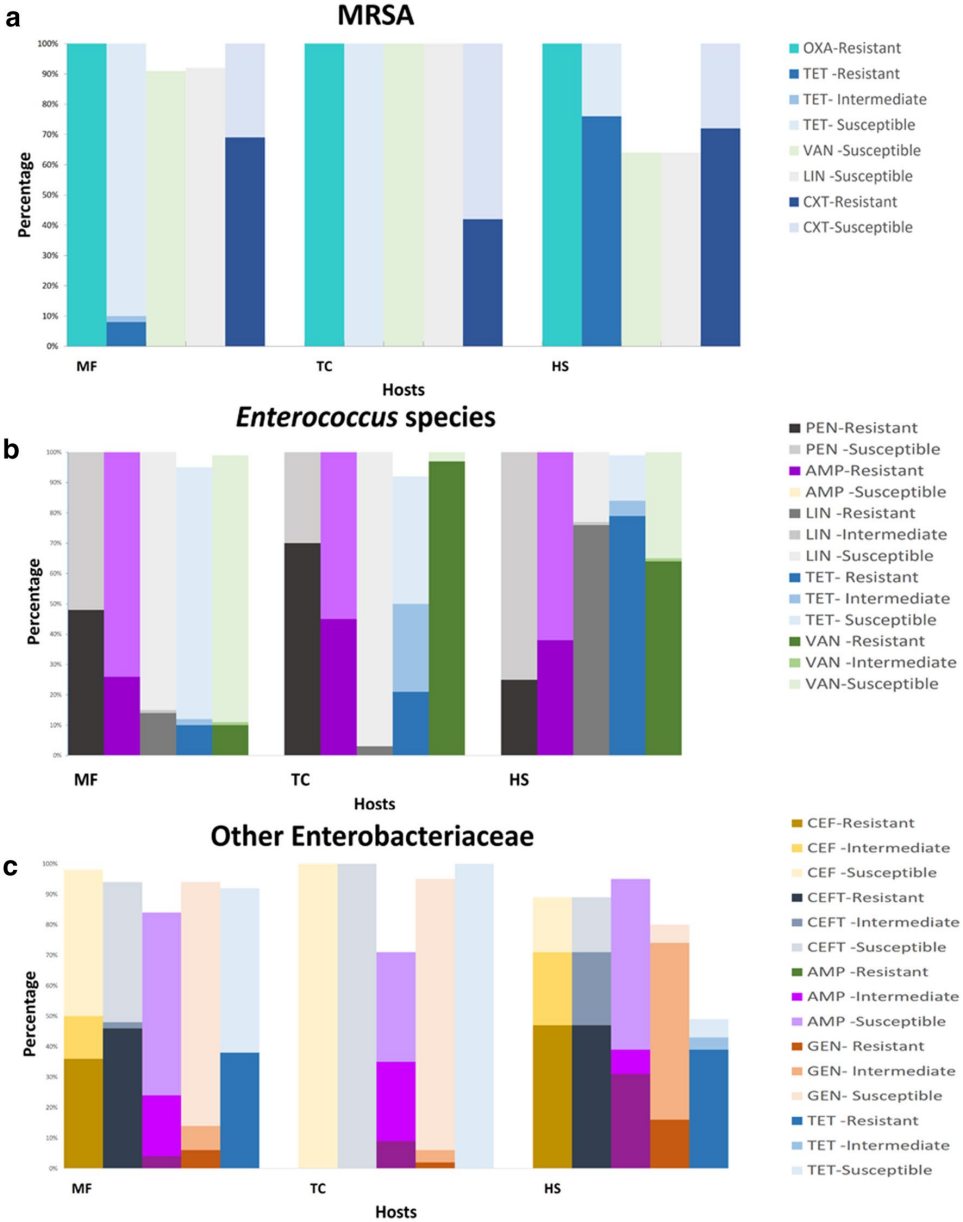
Disk diffusion assay for **a** MRSA isolates; **b**. *Enterococcus* spp. isolates; **c**. Other Enterobacteriaceae isolates. *OXA* oxacillin, *TET* tetracycline, *LIN* linezolid, *CEF* cefoxitin, *VAN* vancomycine, *PEN* penicillin, *AMP* ampicillin, *CEF* cefazolin, *CEFT* ceftazidime, *GEN* gentamicin. Bars that do not add up to 100% contained isolates that do not fall under resistant, intermediate nor susceptible categories based on CLSI

**Fig. 2:**
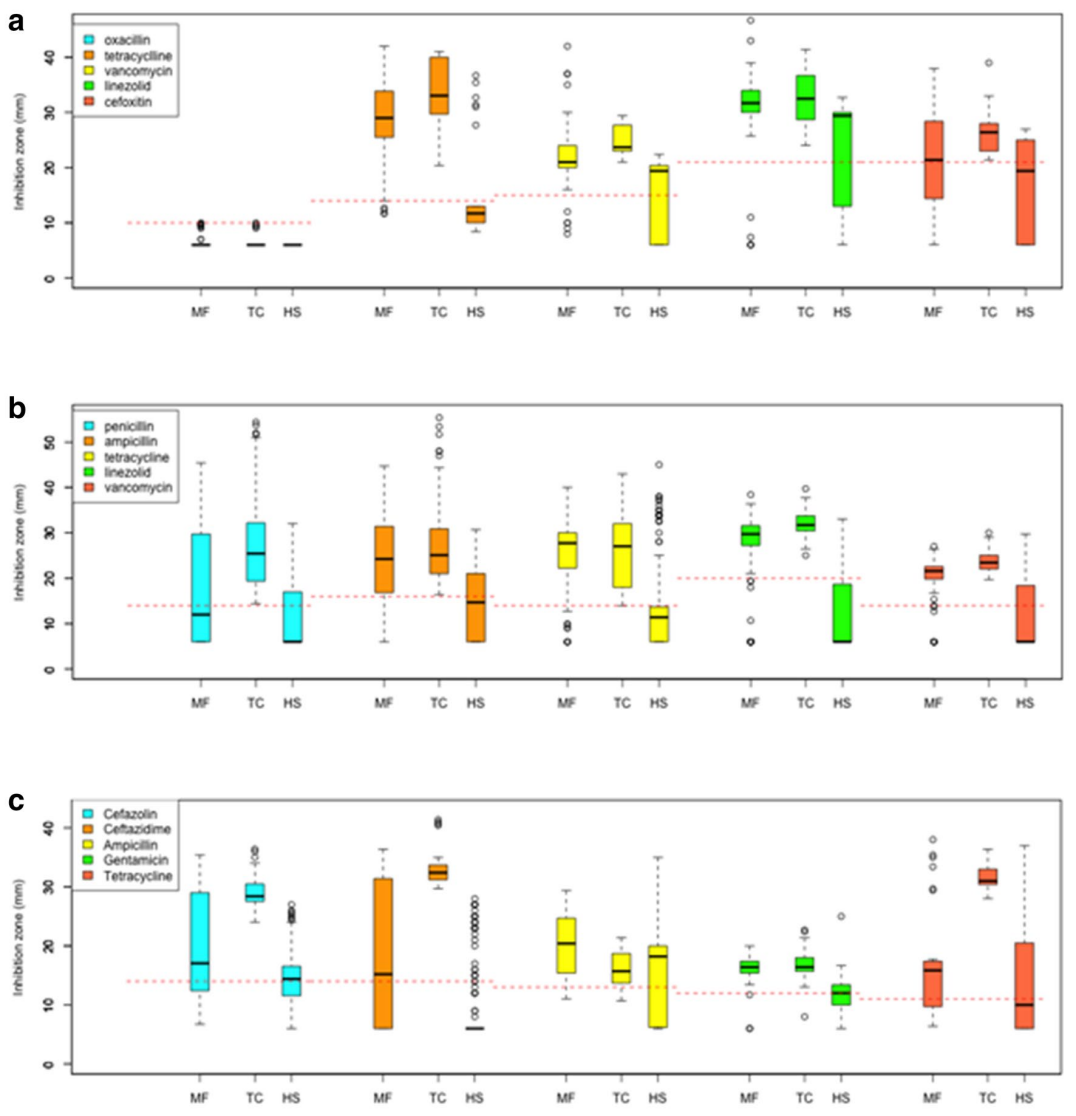
Boxplot of antibiotic resistance profiles inferred based on inhibition zone (mm), **a** MRSA; **b***Enterococcus* spp.; **c** other Enterobacteriaceae

A total of 61, 26 and 25 isolates were obtained from *Macaca fascicularis* (MF, Long-tailed macaque), *Trachypithecus cristatus* (TC, Silvered leaf monkey), and *Homo sapiens* (HS) respectively. The isolates were classified into resistant, intermediate and susceptible based on guidelines from Clinical and Laboratory Standards Institute (CLSI) (Table 1) and expressed in percentage (Fig. 1a). Based on ChromMRSA, all isolates obtained were 100% resistant to oxacillin. Resistance levels for cefoxitin were similar for MF and HS at 70%, compared to TC at 40%. No resistance was observed for linezolid and vancomycin across all hosts. Tetracycline was the most effective antibiotic for bacterial isolates obtained from MF and TC, achieving 100% susceptibility. In comparison, only 25% of the isolates from HS were susceptible to tetracycline. Overall, a distinct antibiotic susceptibility pattern of MRSA across the three hosts was apparent (Fig. 2a).

**Table 1:** Antibiotics and amount impregnated per disk for MRSA, *Enterococcus* spp. and other Enterobacteriaceae

Antibiotic	Disc potency (µg)	Inhibition zone diameter (mm)		
		R	I	S
MRSA				
Oxacillin	1	≤ 17	–	≥ 18
Tetracycline	30	≤ 14	15–18	≥ 19
Linezolid	30	–	–	≥ 21
Cefoxitin	30	≤ 24	–	≥ 25
Vancomycin	30	–	–	≥ 15
*Enterococcus* spp.				
Penicillin	10 units	≤ 14	–	≥ 15
Ampicillin	10	≤ 16	–	≥ 17
Linezolid	30	≤ 20	21–22	≥ 23
Tetracycline	30	≤ 14	15–18	≥ 19
Vancomycin	30	≤ 14	15–16	≥ 17
Other Enterobacteriaceae				
Cefazolin	30	≤ 14	15–17	≥ 18
Ceftazidime	30	≤ 14	15–17	≥ 18
Ampicillin	10	≤ 13	14–16	≥ 17
Gentamicin	10	≤ 12	13–14	≥ 15
Tetracycline	30	≤ 11	12–14	≥ 15

*R* Resistant, *I* Intermediate, *S *Susceptible (CLSI, 2018)

### *Enterococcus* species

**Fig. 3:**
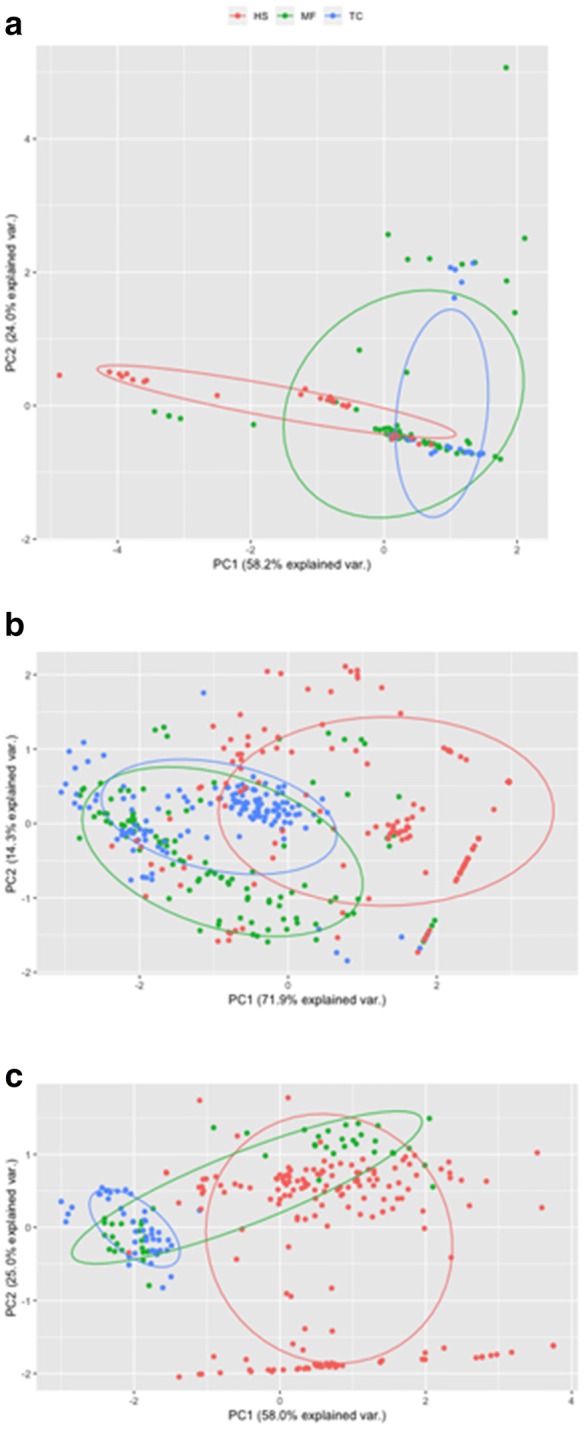
Principal components analysis of the antibiotic resistance profiles. **a** MRSA; **b***Enterococcus* spp.; **c** other Enterobacteriaceae

A total of 108, 141 and 170 isolates were obtained from MF, TC, and HS, respectively. Around 70% of the isolates from TC displayed resistance against penicillin while approximately 50% of MF and 25% of HS isolates showed resistance. Resistance of MF to vancomycin was low at 10% compared to the TC at 97% and HS at 64% (Fig. 1b). HS isolates were 100% resistant to tetracycline (Fig. 2b). HS and TC isolates showed partial resistance to tetracycline. When all 5 antibiotics were compared, TC formed the tightest cluster amongst the three hosts (Fig. 3b).

### Other Enterobacteriaceae

Fifty, 47 and 171 isolates were obtained for MF, TC, and HS respectively. Enterobacteriaceae from MF and HS were resistant to cefazolin and ceftazidime while Enterobacteriaceae from TC showed 100% susceptibility to both antibiotics (Fig. 1c).

### Comparison of fecal bacterial community composition between MF, TC and HS

Significantly lower bacterial richness (Shannon’s and Simpson’s Diversity indices) and evenness were detected in HS in comparison to MF and TC (Additional file 1: Fig. S1). When the distribution of taxa was observed at the phyla level, higher similarity was apparent between MF and TC (the two NHPs) than with HS. For instance, the NHPs showed higher level of Firmicutes and lower level of Bacteroidetes than HS. In contrast, Acidobacteria was found exclusively in HS but not in the MF nor TC (Fig S2A). In genus level, higher *Bacteroides* and *Prevotella* were observed in HS in comparison to the two NHPs (Additional file 2: Fig. S2B).

**Fig. 4:**
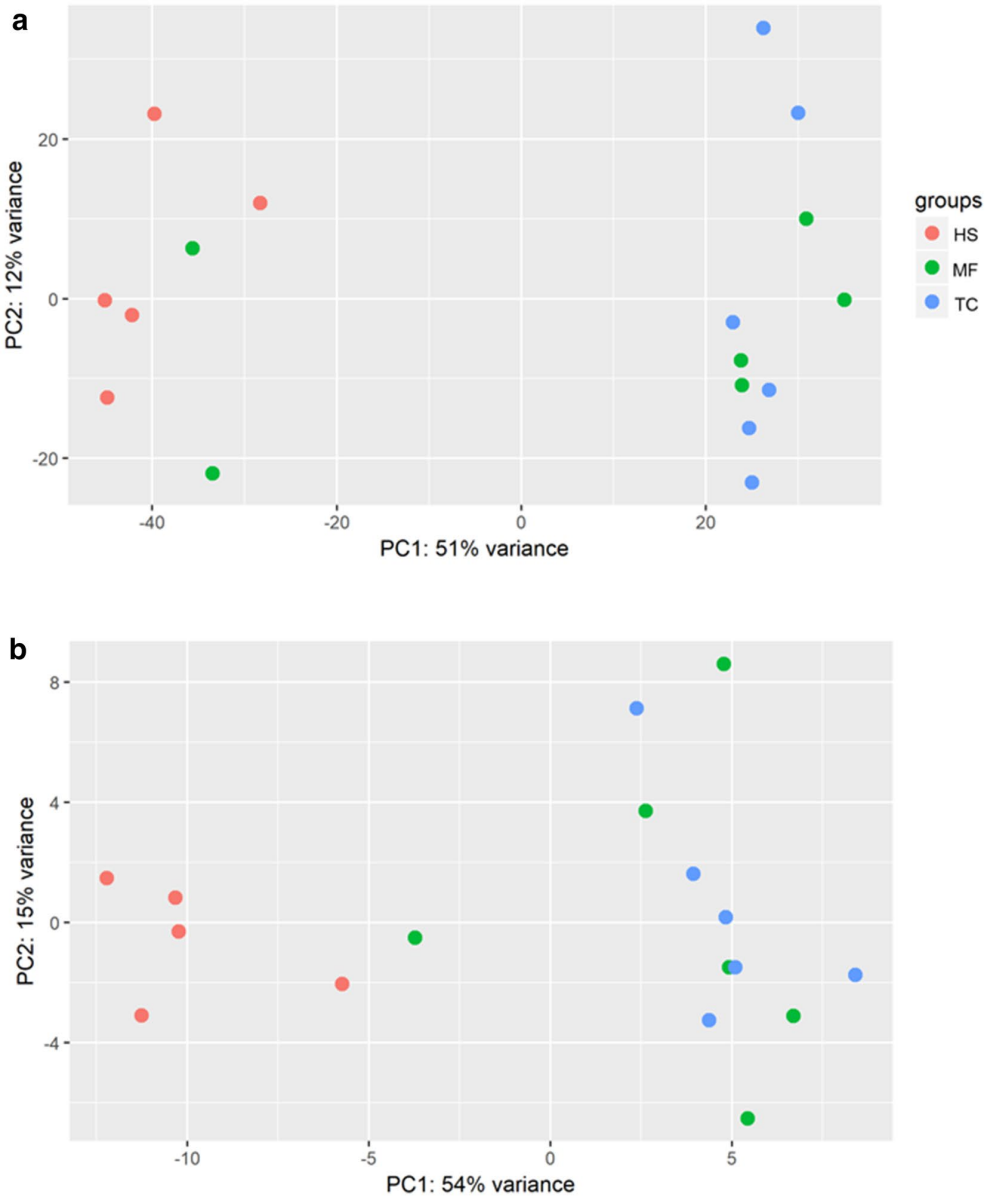
Euclidean distance-based principle coordinate analysis of regularized transformed matrix of **a** OTUs; **b** predicted functional metagenome

**Fig. 5:**
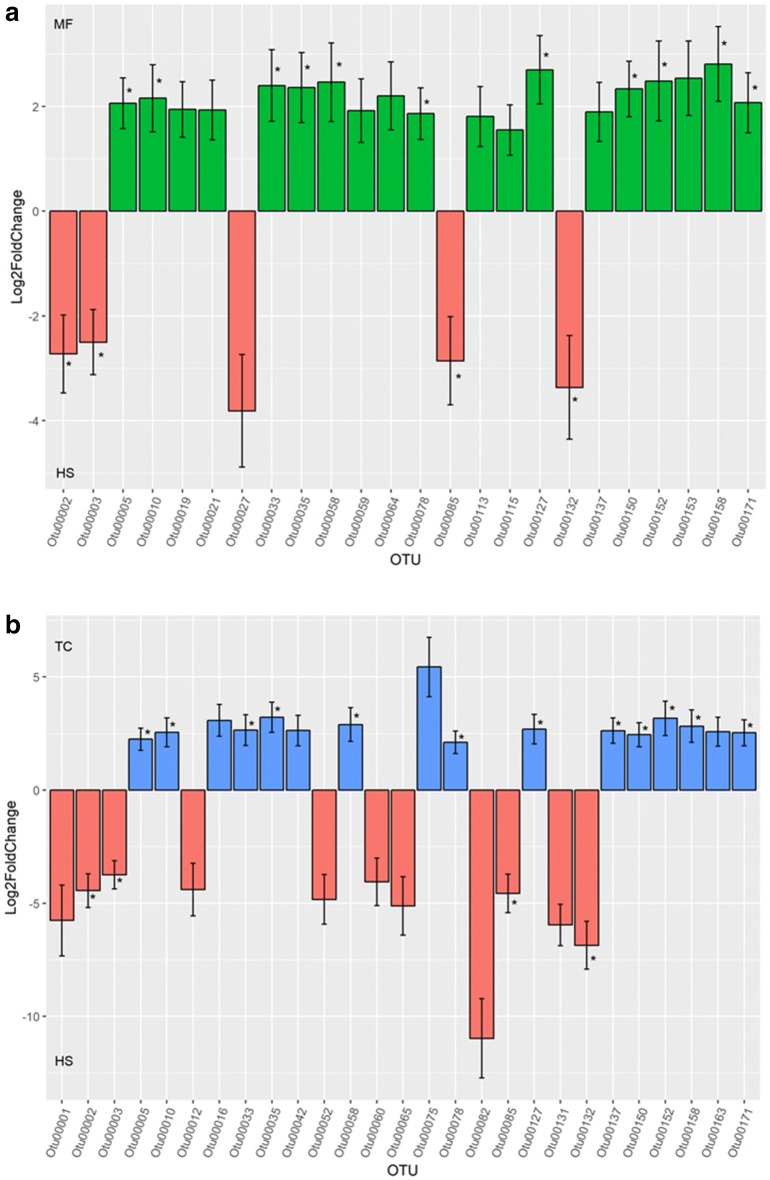
Significantly abundant OTUs derived using negative log binomial model. **a** MF vs HS; **b** TC vs HS

We showed that the distribution pattern of Operational Taxonomic Unit (OTUs) from the two NHPs were more similar to each other than HS (Fig. 4a). Interestingly however, two samples from MF were clustered together with HS along component PC1. Further investigation into the differentially abundant OTUs revealed higher prevalence of *Bacteroides uniformis* (OTU00003), *Bacteroides caccae* (OTU00085), *Bifidobacterium longum* (OTU00132) in HS in comparison to the two primate species. In contrast, NHPs exhibited higher prevalent of OTUs comprising *Christensenellaceae*, *Ruminococcaceae*, *Clostridiales*, and *Oscillospira* from the phylum Firmicutes (Fig. 5, Additional file 3: Table S1).

### Comparison of predicted functional metagenome between MF, TC and HS

**Fig. 6:**
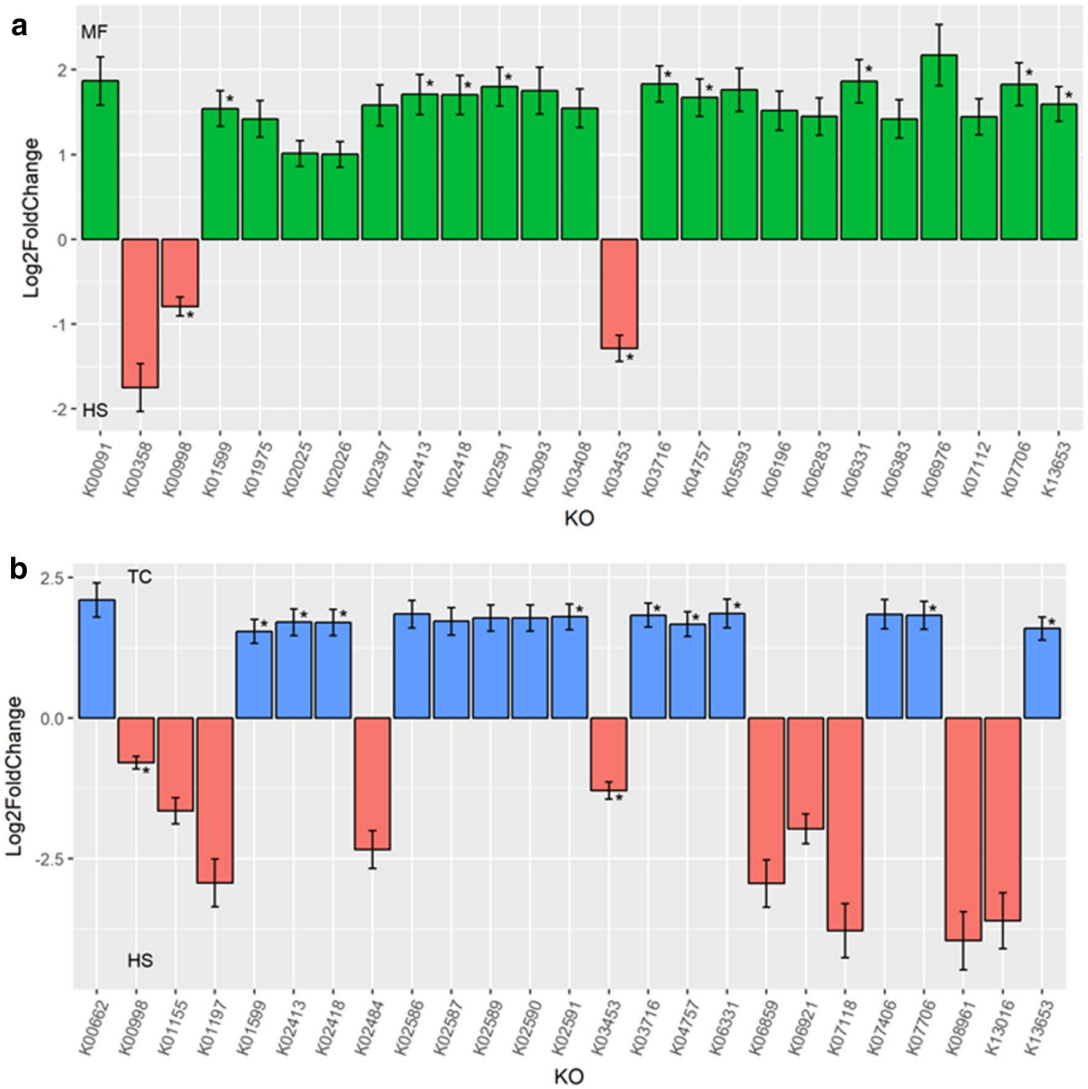
Significantly abundant KEGG orthologs derived using negative log binomial model. **a** MF vs HS; **b** TC vs HS

The distribution of the predicted functional metagenome was illustrated in Fig. 4b. As per the distribution of the bacterial composition, the two NHPs showed higher similarity in predicted functions than HS. Notably, significantly greater abundance of KEGG ortholog related to lipid and carbohydrate metabolism were detected in HS (e.g. K00988 and K06859) (Additional file 4: Table S2). In contrast, the gut community of NHPs harboured more KEGG ortholog for bacterial infection and colonization such as bacterial mobility protein (e.g. K02397, K02413, K02418) and sporulation (K06331) (Fig. 6a, b).

## Discussion

### AMR in the studied hosts

Our findings indicated that isolates from HS has the highest levels of resistance amongst the three hosts. Frequent antibiotic exposure in humans is the most probable cause of antibiotic resistance [13]. We initially postulated that the wild NHPs would exhibit lower levels of antibiotic resistance compared to HS due to limited interaction with humans, as well as absence of antibiotic exposure. Evidence from a previous study showed that NHPs at ecotourism sites might have a higher AMR carriage rate due to constant human presence compared to entirely wild counterparts [14]. The acquisition of antibiotic resistance in wild animals may have a serious consequence on the transmission of antibiotic resistance strains. For instance, the wild animal may act as the carrier of the antibiotic resistance strains. The resistant strains may be further transferred to other members of ecosystems through contact and medium such as soil and water [15].

*Macaca facsicularis*, an endemic species in Asia was found to harbour bacterial species with higher levels of antibiotic resistance than TC. MF is ubiquitous in Peninsular Malaysia, Sabah and Sarawak. Although the species is generally considered to be wild, MF resides at the fringe of the jungle and are well adapted to interact with humans (e.g. scavenging food waste). In addition, tourists commonly feed the macaques and this interaction alters the behaviour of animals [16, 17]. Human-animal interactions may also increase the transmission of zoonotic disease to human especially if the subject had been bitten or scratched by the primates [2, 9, 16]. In comparison, TC which are shy and rarely interact with humans [18] showed the lowest carriage rate of antibiotic resistance strains amongst the three hosts studied. Historically, MF’s have been reported to be in closer contact with people and only in recent years have the TC’s been monitored to be interacting with people in an urban setting [19].

From our study, *Enterococcus* species had the highest level of resistance in all hosts, especially for HS. The high levels of resistance to vancomycin for the isolates from the two NHPs is unexplained and require further investigation. A parallel study on black capuchin monkey in Brazil detected no resistance in the isolated *Enterococcus* spp. while resistance to other antibiotics including rifampicin, tetracycline, erythromycin, nitrofurantoin, chloramphenicol, and ampicillin were associated with the anthropogenic impact [14]. On the other hand, the Enterobacteriaceae showed highest susceptibility to all antibiotics tested. However, this particular finding contract that of Bachiri et al. [20] which showed high prevalence of CTX-M-15 gene in *E.coli* isolated from Barbary macaque, suggesting wide spread resistance to beta-lactam antibiotic among the wild life in Algeria.

*Staphylococcus aureus* is 100% resistant to oxacillin but susceptible to most tested antibiotics. It is interesting to note that resistance to oxacillin is a characteristic of community acquired (CA)-MRSA infection [21]. This might suggest that there is a higher risk of human to primate transfer of resistance *S. aureus* than a zoonotic transfer. In line with this, anthropozoonosis transmission of *S. aureus* was reported from Gambia [22]. Nonetheless, the isolation of unique *S. aureus* ST type in primate suggested that the animal can also be an unappreciated source of MRSA transmission [23].

### Host, gut microbiota and AMR carriage rate

The differences in gut microbiota across the studied hosts coincided with the hosts’ diet. Firmicutes and Bacteroidetes are the main dominant phyla in both NHPs and HS. The NHPs in this study both share some similarly to their diet which mainly consists of plants although MFs are omnivorous. Higher abundance of Firmicutes such as *Ruminococceae* in MF and TC may be associated with higher fiber diet [24]. For instance, genus *Oscillospira* which is able to degrade a wide range of glycans is affiliated with the plant-based diets in humans [25]. Conversely, HS was found to harbour higher proportion of Bacteroidetes (Fig. 6a). Members of Bacteroides was previously found to be prevalent in animal-based diet due to its’ bile-resistant characteristics and the ability to degrade fatty acid into short chain fatty acids (SCFAs) [26]. Interestingly, *Bacteroides caccae* which has the capacity to digest dietary plant polysaccharides was enriched in HS [27].

Overall, the predicted functional metagenome of HS showed greater representation of KEGG orthologs related to functions such as amino acid and fatty acid degradation, as well as bile acid catalysis. In contrast, the gut microbial community of NHPs exhibited more functions related to bacterial colonization and replication, potentially reflecting the ecological process of functionally diverse environmental bacteria establishing within the animal host [28].

It is noteworthy that the lower bacterial diversity in HS (Additional file 1: Fig. S1) correlated with the higher abundance of antibiotic resistant strains (Fig. 1). In addition, the distribution of antibiotic resistance profile (Fig. 3) is consistent with the composition of gut microbial community and functions (Fig. 4) where higher similarity in MF and TC as opposed to HS was observed. It is recognized that the gut bacterial assemblage may control for the colonization of pathogenic bacteria, including those that are antibiotic resistance [29, 30]. As such, while the lower presentation of antibiotic resistant isolates in the primate may be explained by lower exposure to antibiotic, the native bacterial composition may also play a role in preventing the establishment of the viable colony of the resistant strains in the gut. Furthermore, the gut microbiota may be the reservoir for transfer of antibiotic resistance gene via horizontal gene transfer [31]. As NHPs harbour different gut microbiota in comparison to humans, the transmission of antibiotic resistant pathogens may facilitate transfer of resistance to conventionally susceptible bacterial taxa.

## Conclusion

Activities such as feeding, petting and photography in ecotourism narrows the gap between humans and NHPs, inevitably increasing the potential for the spread of pathogens specifically antibiotic resistant pathogens. In this study, the isolates obtained from the two studied NHPs were found to exhibit lower antibiotic resistance in comparison to the human subject. The results might indicate low rate of transfer from human to primates in the nature park but still a likely possibility. We speculate that the low carriage rate may also be contributed by differences in gut microbial composition, which control the colonization of the resistant pathogens. We argue that provisioning of food to NHPs could alter gut microbiome in due course and over time affect carriage rates. A greater awareness about pathogen transfer to tourists and residents is important to avoid acquiring pathogens but also spreading it which could impact wildlife in the future. We advocate that clear guidelines at tourist sites where unmonitored human-animal interaction takes place should be available to prevent the risk of anthropozoonosis as well as zooanthroponosis.

## Methods

### Ethical approval

Ethics approval for collection of human and non-human primate sampling was obtained from the Perdana University-Internal Review Board (PU-IRB) and was granted under IRB ID: PU IRBHR0088.

### Study location

Kuala Selangor Nature Park (KSNP), and Bukit Melawati in Kuala Selangor, Malaysia were selected as the study sites due to its varied landscape of natural parks as well as popular tourist destination where people interact with wildlife. The ecotourist area is adjacent to a Bukit Melawati residential community equipped with hostels, shops and schools. The park has been in operation since 1987 and covers over 200 hectares of coastal land, which is mainly mangrove swamps.

### Study hosts

**Fig. 7:**
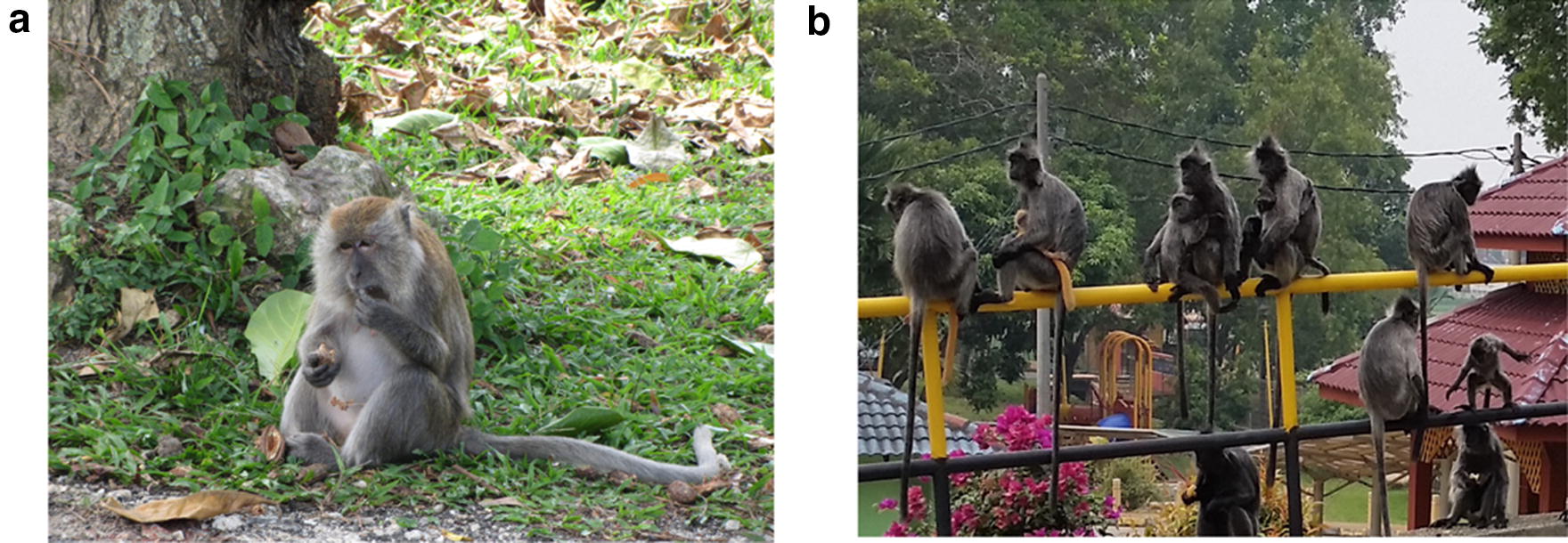
The non-human primates in the study. **a***Macaca fascicularis* (MF) **b***Trachypithecus cristatus* (TC)

The KSNP is a habitat for a significant number of NHPs, amongst which are long-tailed macaques (*Macaca fascicularis*) and silver leaf monkeys (*Trachypithecus cristatus*) (Fig. 7). The non-human primates used in this study. A: *Macaca fascicularis* (MF), B: *Trachypithecus cristatus* (TC)). The park was divided into different zones based on the locations where the NHPs were usually found. Zones A, B, C, and H were the high-interaction zones between NHPs and humans, while the rest (D-K) were medium- and low- interaction zones.

### Sample location

A total of 55 fresh fecal samples were collected, which were made up of 20, 20 and 15 samples of MF, TC and human (HS), respectively. NHPs samples were collected freshly upon defecation and consisted of both male and female samples. MF are omnivorous and TC are predominantly folivorous. However, both species were seen to be provisioned with similar human food like bread, nuts, chips etc. Efforts were made to avoid repeat sampling from the NHPs during the same day. HS samples were collected from healthy volunteers over the age of 18 who were visiting the Ecotourism Park or were residents in the neighbouring area at Kuala Selangor. Four samples were from tourist and the remaining 11 were from residents. Seven of the samples were from people with the age range of 40–60 years old, the remaining 8 were from those between 19 and 28 years old. The type of diet consumed by the HS were not documented. All fecal samples were collected between 10 in the morning and 6 in the evening within a period of 8 months to minimize diurnal variation between February to September 2016. The samples were placed in fecal containers and labeled. The samples were placed in an ice box immediately after collection to preserve bacterial content in the samples. Samples were transported to the lab daily in an ice box and prior to storage, each fecal sample was weighed to 0.1 g and aliquoted into 1.5 mL Eppendorf tubes. The samples were then stored at − 20 °C in the freezer until further use.

### Isolation and enumeration of bacteria

Nine hundred μL of 0.9% sodium chloride (Kollin, USA) was added to 0.1 g of the fecal sample which had been aliquoted earlier. The mixture was homogenized using a vortex mixer for 10 s. Dilutions up to 10^–1^, 10^–2^, 10^–3^, 10^–4^ and 10^–5^ solution were prepared through ten-fold serial dilutions. One hundred μL of each dilution was then inoculated on Tryptone Soya Agar, TSA (Oxoid, United Kingdom) in triplicates under aseptic condition. The plates were then incubated for 18–24 h at 37 °C. Approximately 30 to 300 colonies were selected for replica plating.

### *Staphylococcus* spp.

Sample was plated onto Mannitol Salt Agar, (MSA), (Oxoid, United Kingdom) for the selection of *Staphylococcus* spp., then the MSA plates were subsequently plated onto ChromMRSA (Chromagar, Oxoid, United Kingdom) for isolation of methicillin-resistant *Staphylococcus aureus* (MRSA). These plates were incubated for 18–24 h at 37 °C before enumeration of the colonies was carried out.

### Enterobacteriaceae

*Enterococcus* spp. was isolated using Slanetz and Bartley Medium (SBM) (Oxoid, United Kingdom) while the isolation of *other* Enterobacteriaceae was carried out using Eosin Methylene Blue Agar (EMBA) (Oxoid, United Kingdom). The SBM plates were incubated for 48 h at 30 °C and EMB plates were incubated for 18–24 h at 37 °C. Subsequently, enumeration of the colonies was carried out.

### Antibiotic susceptibly test

Antibiotic susceptibility tests were carried out using agar disc diffusion method using antibiotic impregnated discs (CLSI, 2018). A bacterial lawn was established using a sterile cotton swab whereby the concentration of the bacteria was adjusted according to 0.5 MacFarland (1.5 × 10^8^ Colony forming units (CFU/mL) Then, discs containing antibiotics were placed onto the agar plates using the incubation condition as described in the method used for bacterial isolations. The diameter of the zone of inhibition was recorded for all antibiotics post-incubation. Distilled water was used as a negative control and the experiment was carried out in triplicates. *E. coli* ATCC 25922 and *S. aureus* ATCC 25923 were used as control strains in the disc diffusion test.

MRSA, *Enterococcus* spp. and other Enterobacteriaceae isolates from ChromMRSA, SBM and EMBA were tested for antibiotic resistance using specific range of antibiotics (Table 1). For instance, MRSA was tested with oxacillin, tetracycline, cefoxitin, linezolid and vancomycin. For *Enterococcus* spp., ampicillin, tetracycline, vancomycin, penicillin and linezolid were used. Lastly, ampicillin, gentamicin, tetracycline, cefazolin and ceftazidime were tested on other Enterobacteriaceae. All reference ranges were adapted from CLSI 2018.

Twenty μL of antibiotics with defined concentration were impregnated into the 6 mm disks (Oxoid, United Kingdom) and classified into resistant, intermediate and susceptible based on CLSI (2018). Putative MRSA, enterococci and other Enterobacteriaceae isolates were subjected to disc diffusion testing using Mueller–Hinton Agar (MHA). Briefly, all isolates were adjusted to 0.5 MacFarland (1.5 × 10^8^ CFU/mL) before the disks were placed onto MHA. The diameter of inhibition zones for each antibiotic were measured after 24 h of incubation at 37 °C for detection of antibiotic resistance in *Enterococcus* spp. and other Enterobacteriaceae. For detection of MRSA, the plates were incubated for 24 h at 30 °C [32].

### DNA extraction

Total genomic DNA from the fecal samples was extracted using the QIAamp stool DNA mini kit according to manufacturer’s protocol (Qiagen, Valencia, CA). The extracted DNAs were purified and quantified using Nanodrop (USA) at 260 nm and 280 nm prior to 16S sequencing using Illumina Miseq.

### Amplicon sequencing of 16S rDNA gene

In total, 17 samples were obtained from the three hosts (HS, MF and TC) i.e. six samples for MF and TC and five for HS. All sequences obtained were submitted to GenBank under BioProjectID: PRJNA590002. The taxonomic diversity presented in microbial communities was analysed through sequence variation in the 16S ribosomal RNA (rRNA) gene. A total of 2,262,680 raw reads generated from Illumina paired-end sequencing were processed and filtered using Mothur version 1.39.5 [33]. The sequences were clustered and assigned to operational taxonomic unit (OTUs) using the reference SILVA SEED Database Release 132 [34]. Chimeric sequences were identified and removed using VSEARCH, which was implemented within the Mothur pipeline. The final dataset consisted of 669 OTUs from 1,045,777 sequences, with mean length of 418 bp. The data was rarefied to equal depth of 25,273 sequences per sample. Alpha diversity was compared using Shannon’s Diversity Index, Simpson’s Diversity Index and Pielou’s Evenness. In addition, bar charts were constructed using phyloseq package [35] to display the proportional differences in genus and phylum across groups.

Overall differences in bacterial composition between hosts (beta diversity) were evaluated using Permutational Multivariate Analysis of Variance (PERMANOVA). Further, taxa showing significant differences in abundance between host species were identified using negative log binomial model implemented in DeSeq2 R package [36].

## Supplementary information

**Additional file 1: Fig. S1.** Comparison of Alpha Diversity Indices across MF, TC and HS.

**Additional file 2: Fig. S2.** Distribution of A) Phylum-based and B) Genus-based bacterial composition from MF, TC and HS.

**Additional file 3: Table S1.** The taxonomic identity of significantly different OTUs detected using negative binomial log model.

**Additional file 4: Table S2.** The identity of significantly different KEGG orthologs detected using negative binomial log model.

## Data Availability

The datasets used and/or analysed during the current study are available from the corresponding author on reasonable request. All data generated or analysed during this study are included in this published article (and its additional files).

## Abbreviations

NHP: Non-human primates
MF: *Macaca fascicularis*
TC: *Trachypithecus cristatus*
HS: *Homo sapiens*
AMR: Antimicrobial resistance
KSNP: Kuala Selangor Nature Park
MRSA: Methicillin-resistant *Staphylococcus aureus*
CA-MRSA: Community acquired-methicillin-resistant *Staphylococcus aureus*
AHI: Animal human interaction
CLSI: Clinical & Laboratory Standards Institute
MSA: Mannitol Salt Agar
SBM: Slanetz and Bartley Medium
EMBA: Eosin Methylene Blue Agar
