# Supplementary figures and images for: Association of antimicrobial resistance and gut microbiota composition in human and non-human primates at an urban ecotourism site
Source: Gut Pathog. 2020 Mar 10;12:14. doi: 10.1186/s13099-020-00352-x (PMC7063749; doi:10.1186/s13099-020-00352-x)

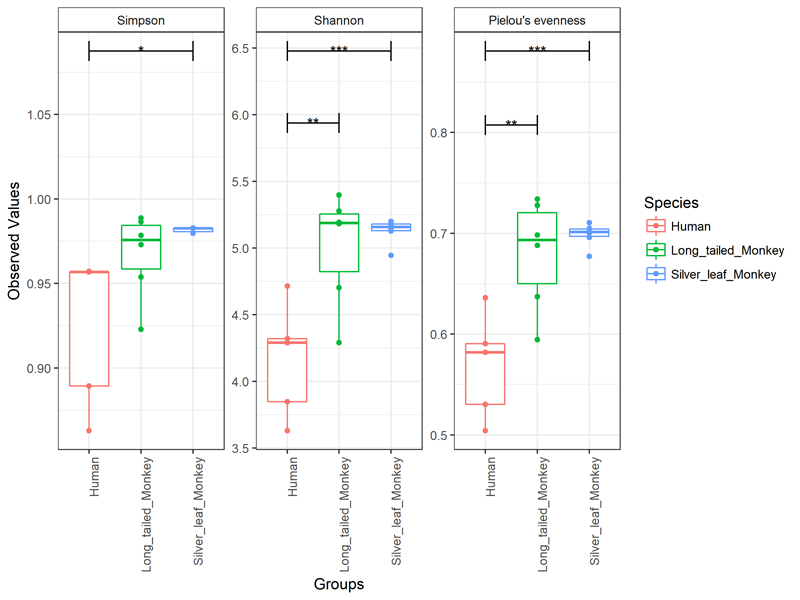

Supplement: Supplementary file 1 — Additional file 1: Fig. S1. Comparison of Alpha Diversity Indices across MF, TC and HS. [file 13099_2020_352_MOESM1_ESM.bmp]

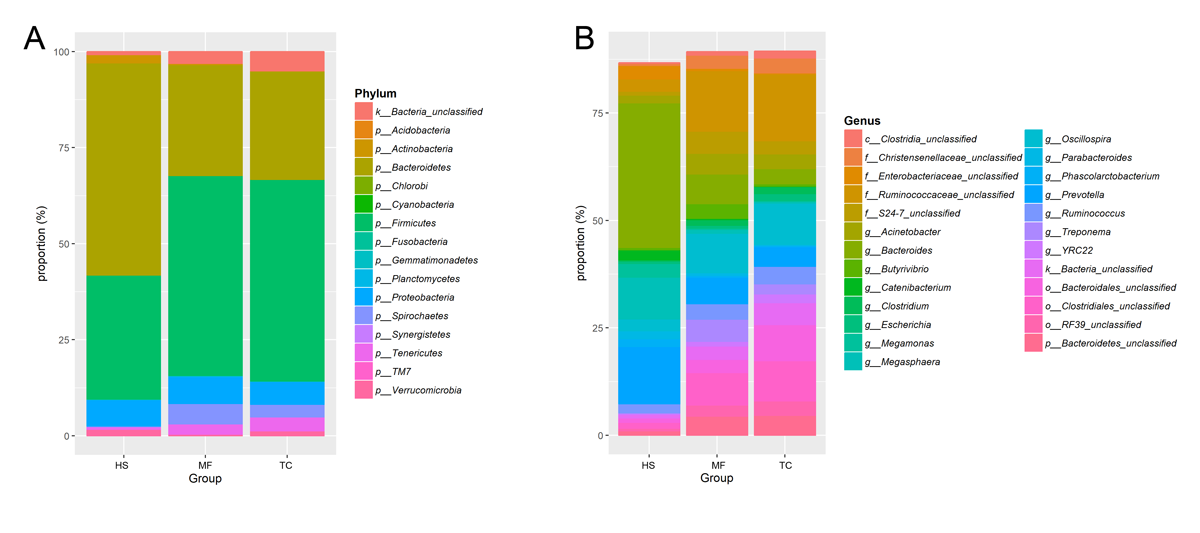

Supplement: Supplementary file 2 — Additional file 2: Fig. S2. Distribution of A) Phylum-based and B) Genus-based bacterial composition from MF, TC and HS. [file 13099_2020_352_MOESM2_ESM.bmp]
